# *Lawsonia intracellularis*: Revisiting the Disease Ecology and Control of This Fastidious Pathogen in Pigs

**DOI:** 10.3389/fvets.2018.00181

**Published:** 2018-08-09

**Authors:** Anbu K. Karuppannan, Tanja Opriessnig

**Affiliations:** ^1^Department of Veterinary Diagnostic and Production Animal Medicine, College of Veterinary Medicine, Iowa State University, Ames, IA, United States; ^2^The Roslin Institute and The Royal (Dick) School of Veterinary Studies, University of Edinburgh, Midlothian, United Kingdom

**Keywords:** *Lawsonia intracellularis*, pigs, control, gut microbiome, nutrition

## Abstract

*Lawsonia intracellularis* is an anaerobic obligate intracellular bacterium infecting the small intestine and infrequently also the large intestine of pigs and other animals including hamsters and horses. The infection is characterized by proliferation, hemorrhage, necrosis, or any combination commonly referred to as “ileitis,” affecting the health and production efficacy of farmed pigs. Despite decades of research on this pathogen, the pathogenesis and virulence factors of this organism are not clearly known. In pigs, prophylaxis against *L. intracellularis* infection is achieved by either administration of subtherapeutic levels of in-feed antibiotic growth promoters or vaccination. While the former approach is considered to be effective in *L. intracellularis* control, potential regulations on subtherapeutic antibiotics in many countries in the near future may necessitate alternative approaches. The potential of manipulating the gut microbiome of pigs with feed ingredients or supplements to control *L. intracellularis* disease burden is promising based on the current understanding of the porcine gut microbiome in general, as well as preliminary insights into the disease ecology of *L. intracellularis* infection accrued over the last 30 years.

## Introduction

Enteric diseases in pigs, especially around weaning, are very common and often of infectious nature (1). While viruses play a main role in young pigs, bacteria and protozoa are often contributing to diarrhea and related clinical signs and subsequent reduction of average daily gain in growing pigs. Among economically important bacteria, *Lawsonia intracellularis* (*L. intracellularis*) is prevalent worldwide and the infection manifests in pigs in two clinical presentations (2). The chronic proliferative form of *L. intracellularis* infection, described as proliferative enteropathy (PE) or porcine PE (PPE), is commonly observed in weaned and growing pigs less than 4 months of age. It is associated with decreased weight gain and low mortality due to proliferation and thickening of the ileum and the proximal colon. Acute *L. intracellularis* infection is known as proliferative hemorrhagic enteritis (PHE), and is characterized by intestinal hemorrhages and sudden death, usually occurring in mature pigs older than 4 months (2).

### Initial clinical characterization and isolation of *L. intracellularis*

**Figure 1:**
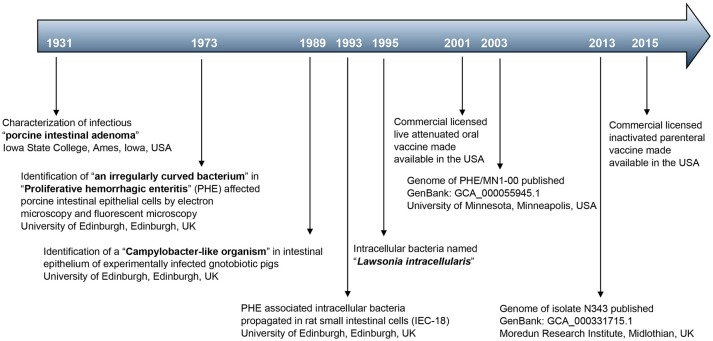
Major milestones in *L. intracellularis* research and control.

Occurrence of intestinal adenoma in swine, described as “degeneration of the epithelium, formation of adenomatous growths and transition from goblet cells to undifferentiated non-mucin-containing cells in the ileum and colon” was first documented in 1931 (Figure 1) by investigators from the Department of Veterinary Investigation, Iowa State College, Ames, Iowa, USA (3). The authors describe experimental reproduction of this disease in 12 pigs, by “feeding intestinal contents and scrapings from the mucosa of infected swine,” which manifested as acute dysentery and proliferative epithelial lesions in the intestines (3). This seminal work proved the infectious nature of “porcine intestinal adenoma” now known as PE. In 1973, researchers from the Royal (Dick) School of Veterinary Studies, Edinburgh, UK, identified with electron microscopy “an irregularly curved bacterium” in affected intestinal epithelial cells of pigs with PHE (4). Staining of affected intestinal epithelium cells with fluorophore labeled serum from affected pigs revealed specific signals in their apical cytoplasm (4, 5). In 1989, the same research group was able to identify Campylobacter-like organisms in the cytoplasm of enterocytes in gnotobiotic pigs experimentally infected with intestinal mucosa homogenate from a pig with PHE (6). They suggested that these organisms enter the enterocytes and multiply intracellularly in affected tissues after invasion. Axenic culture of the intracellular organism in conventional culture medium could not be achieved (6). A disease similar to PHE, transmissible ileal hyperplasia (TIH), was known in hamsters since 1965 (7). Similar to PHE, light and fluorescent microscopic observations in affected hamsters showed curved rod-shaped bacteria located in the apical cytoplasm of immature crypt epithelial cells in the ileum (5, 7–9). The TIH associated bacteria were thought to replicate intracellularly, and appeared to accumulate in hyperplastic epithelial cells (5). Oral inoculation of hamsters with TIH associated intracellular bacteria, propagated in intestine 407 cells (ATCC® CCL-6™), resulted in clinical disease (10). In 1993, the bacteria associated with PE/PHE in pigs were successfully propagated in rat small intestinal cells (IEC-18; ATCC® CRL-1589™) under reduced oxygen level of 8% (11). Infected cells were noted to spread the intracellular bacteria to their daughter cells during cell division, which was not affected by the presence of neomycin in the cell culture media. The intracellular organism appeared 0.3 μm in diameter and 1.0 μm in length, and was curved or rod shaped with a trilaminar outer membrane (11). The cell culture propagated intracellular organisms, devoid of any other bacteria including chlamydia, were able to reproduce PHE in conventional pigs by oral infection (12). Interestingly, gnotobiotic pigs could not be infected with the same inoculum stock (12). However, in earlier experiments gnotobiotic pigs could not only be successfully infected with intestinal homogenate from naturally infected pigs, but also developed clinical PHE (6). Pure IEC-18 cell cultures of the intracellular bacterium obtained from naturally PHE affected pigs were used to experimentally infect hamsters via the oral route (13). Infected hamsters developed proliferative enteritis showing the common etiology of the proliferative lesions (13). The bacterium, designated *ileal symbiont intracellularis (IS intracellularis)*, was characterized as Gram-negative and acid-fast positive using modified Ziehl Neelsen staining (14). The 16S rRNA sequence of *IS intracellularis* showed the highest similarity to *Desulfovibrio desulfiricans*, a sulfate reducing bacteria found in anaerobic niches (14). Further confirmation of phenotypic and genotypic characters led to the renaming of *IS intracellularis* to *L. intracellularis*, in honor of Dr. G. H. K. Lawson who first identified the intracellular bacteria in 1973 (15). *L. intracellularis* is classified into the family *Desulfovibrionacea* and phylum Proteobacteria (16). The organism was initially thought to have no flagellum. However, later observations of *L. intracellularis* outside of cells showed a unipolar flagellum used to achieve a darting motion in the extracellular environment (16, 17). Natural or experimental observation of *L. intracellularis* infection has been observed in horses, rats, rabbits, ferrets, foxes, dogs, sheep, deer, ratites, and non-human primates (16). Under natural conditions*, L. intracellularis* is spread by the fecal-oral route of infection (18). The organism is known to survive in fecal material at 15°C for up to 2 weeks (19).

### Pathogenesis of *L. intracellularis* infection

**Figure 2:**
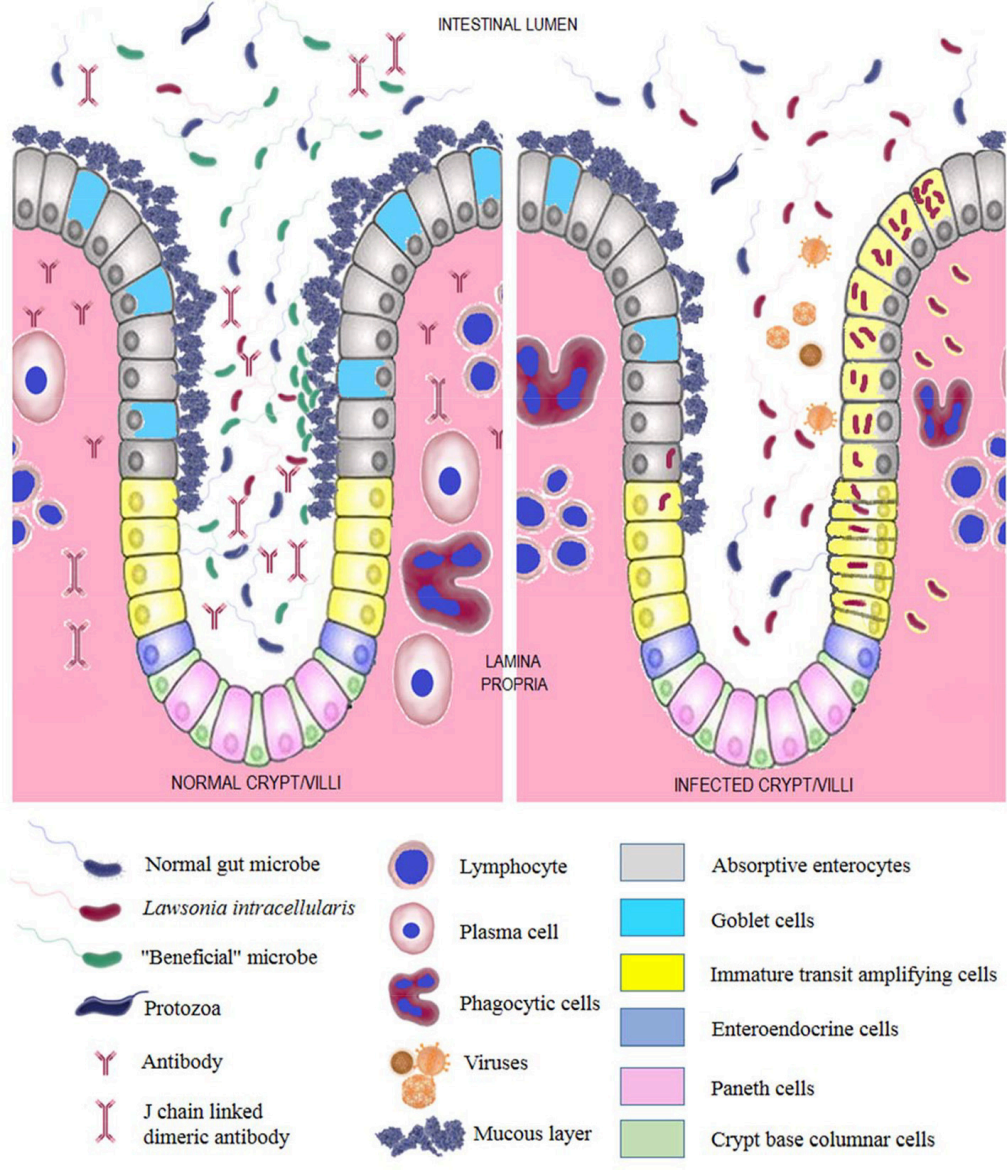
*L. intracellularis* infection and gut ecology. Normal gut microbiome, beneficial microbes promoted by prebiotics and probiotics, adequate mucous layer and protective antibodies confers resistance to *L. intracellularis* infection (left). A microbial ecology representative of “dysbiosis” characterized by depleted or altered normal microbiota, inadequate intestinal mucus layer and low level of protective antibodies is observed in *L. intracellularis* infection (right).

Clinical signs of *L. intracellularis* infection in pigs are often characterized by acute diarrhea of varying severity and occasional black tarry feces which can progress to watery diarrhea with frank blood (2). Additional common observations include pallor, weakness, and rapid death. Subclinical disease, often noticed as variation in pig size, may be accompanied by sporadic diarrhea, decreased growth rates, and potentially anorexia and apathy (2). Monitoring of individual weight gain may help in early identification of subclinical *L. intracellularis* infection. Experimental oral inoculation of pigs with *L. intracellularis* leads to establishment of infection of the intestinal epithelium after 3–5 days, with visible gross lesions appearing after 11–15 days which may be present for another 14 days before resolution (16, 17, 20, 21). The organism is shed in feces from around 7 days after infection, and then for up to 12 weeks (2). Diarrhea starts at 9 days after infection and is observed for 21 days. *L. intracellularis* antigen is observed in the epithelial cells of the distal small intestine and the proximal large intestine until 28 days post-infection. Macroscopic lesions consisting of thickening of the intestinal wall with a ridged or cerebriform appearance, often referred to as “hose like thickening,” are primarily observed in the terminal ileum, but commonly extend up to 60 cm proximal of the ileocecal valve into the first third of the proximal colon (2, 22). Bloody intestinal content, occasionally with frank blood clots, may be present. In severe cases the lesions may be present in the jejunum, the cecum, and distal colon. Occasionally, lesions can be limited to the large intestines (22). Histologic lesions may be infrequently found in the spiral colon and also the rectum. The crypt epithelial cells, especially those at the crypt-villus junction (Figure 2), are thought to be infected first (20). Infected epithelial cells spread the infection as they divide and migrate. Marked hyperplastic proliferation of immature crypt epithelial cells can be observed. Infected enterocytes accumulate the organisms in the apical end (23). Heavily infected cells are swollen and form protrusions, which are thought to mediate bacterial spread into the lumen when microvilli are lost (23). As the infection progresses, *L. intracellularis* organisms can also be observed in macrophages located in the lamina propria, even after the clearance of *L. intracellularis* antigen in epithelial cells (20, 22). Therefore, macrophages are thought to play a role in the spread of the infection (20, 22). Apoptotic epithelial cells and macrophages appear in the healing stages of the infection (23). It is now understood that apoptosis is not involved in the pathogenesis of PE, even though contradicting views were reported earlier (16, 21). A recent study suggests that a combination of Notch-1 signaling and disruption of the β-catenin/Wnt pathway may be associated with immature crypt cell proliferation (24). Failure of crypt cells to differentiate into goblet and absorptive cells results in a severe decrease of MUC2 glycoprotein in the affected epithelium at the peak of infection (24). Among studies investigating the host cell response to *L. intracellularis* infection, a combined laser capture microdissection and RNAseq was performed recently (25–28). Research utilizing this meticulous approach identified that infected enterocytes upregulate genes associated with the G_1_ phase of the cell cycle, resulting in activation of transcription and protein biosynthesis (27). With the exception of copper uptake transporters, genes associated with nutrient acquisition were down regulated in infected enterocytes, which is thought to result in malabsorptive diarrhea (27). Factors required for adherence, entry and propagation of *L. intracellularis* in host cells, including host cell proliferation and subsequent spread of the bacterium to adjacent cells, are still unknown. Host adaptation of porcine and equine *L. intracellularis* isolates is observed and their ability to cause pathogenesis in heterologous hosts is limited (29). Interestingly, porcine and equine host adapted *L. intracellularis* strains can infect and induce pathogenesis in hamsters and rabbits, respectively (30). Conversely, porcine isolates are not pathogenic to rabbits and equine isolates are not pathogenic to hamsters (30).

### *L. intracellularis* genome

Full genome sequences of two pathogenic pig isolates of *L. intracellularis* (Table 1) are published to date (31, 32). The genome consists of a 1.46 Mb chromosome and three plasmids of 0.03, 0.04, and 0.19 Mb (32). Between the two genomes, eight single-nucleotide polymorphisms (SNPs) and 70 insertion/deletions (indels) in intragenic regions have been noted. In addition, 16 SNPs (3 synonymous, 13 non-synonymous) and 20 indels were identified in intergenic regions. A third genome of an equine isolate of *L. intracellularis* (Table 1) was published in 2017 (32). An 18 kb prophage-associated genomic island specific for porcine isolates has been identified by comparative analysis of genomes (33). Since this genomic island is not found in *in vitro* cultured *L. intracellularis* PHE/MN1-00 after 60 passages (non-pathogenic) nor in equine or rabbit isolates, it may not be required for pathogenicity in pigs but rather be a feature of adaptation to the porcine host (33). Genomic comparison of pathogenic passage 10 and non-pathogenic passage 60 of isolate PHE/MN1-00 shows only four SNPs in the chromosome and one SNP in plasmid C, apart from the absence of the 18 kb prophage associated genomic island. The lack of prominent differences at the genomic level suggests an epigenetic regulation of virulence-associated genes in the course of cell culture adaptation (33, 34). Comparative transcriptome profiling of passages 10 and 60 of isolate PHE/MN1-00 revealed 401 genes that were exclusively expressed by the passage 10 bacterium and 319 genes that were expressed at both passage levels (35). Interestingly, all plasmid A genes were repressed in the non-pathogenic passage 60; these genes are implicated in membrane transport, adaptation and stress response apart from other biological functions, and included a few novel genes with unknown functions. The isolate PHE/MN1-00 is predicted to encode 1419 proteins [National Center for Biotechnology Information (NCBI), Genome Assembly and Annotation report, NCBI reference sequence NC_008011.1] and the isolate N343 [GenBank accession numbers CP004029 (chromosome) and CP004030, CP004031 and CP004032] is predicted to encode 1339 proteins (31). Genes related to adhesion, invasion, virulence are identified in the genome. Specifically, genes related to type III secretion systems and type V autotransporter have been noted (31). Only a few of these genes/proteins have been experimentally analyzed. The genome also shows genes related to adaptation to an intracellular life style, such as presence of a putative ATP/ADP translocase gene, absence of genes involved in the tricarboxylic acid cycle, and synthesis of specific amino acids (31).

**Table 1:** List of complete genomes of *L. intracellularis*.

**Genbank accession**	**Isolate**	**Host**	**Year**	**Institution**
GCA_000055945.1	PHE/MN1-00	Pig	2003	University of Minnesota, Minnesota, USA
GCA_000331715.1	N343	Pig	2013	Moredun Research Institute, Midlothian, UK
GCA_001975945.1	E40504	Equine	2017	University of Minnesota, Minnesota, USA

The *L. intracellularis* genome was first sequenced in 2003, but so far little-to-no information is available on the encoded proteins. In 1998 it was found that the *groES/EL* operon of *L. intracellularis* encodes a 60 K Da GroEL protein which reacted with *L. intracellularis* specific antisera raised in rabbits (36). The *L. intracellularis* surface antigen A (LsaA) is expressed in cell culture and in infected ileal tissue, and was identified using degenerate primers (37). Monoclonal antibodies against LsaA were able to block the *in vitro* infection of intestinal epithelial cells by *L. intracellularis*. The gene for ATP/ADP translocase of *L. intracellularis*, a protein known to be involved in energy parasitism by intracellular pathogens, was identified based on homology, and its functionality was verified by heterologous expression in *E. coli* (38). In another study, *L. intracellularis* secretion component N (*lscN*), *lscO*, and *lscQ* genes, a family of virulence genes found in many bacteria which together encode a putative type 3 secretion system, were found to be expressed in infected pig intestinal epithelial cells (39). Further, recombinant LscQ was found to react with serum from infected and vaccinated pigs (39). Another protein, the *L. intracellularis* autotransporter protein A (LatA), was identified based on mass spectrometric analysis of cell culture expressed *L. intracellularis* proteins that reacted with infected pig sera (40). LatA was shown to specifically react to sera from infected pigs. Another mass spectrometry study utilizing a shotgun proteomics approach identified five outer membrane proteins of *L. intracellularis*, of which two were antigenic and reacted with infected pig serum (41). A putative hemolysin of *L. intracellularis* named LhlyA has been examined at a preliminary level (42).

## Control of *L. intracellularis*

### Disinfectants

Various commercially available disinfectants based on quaternary ammonium compounds (Roccal-D Plus®, DC&R®, Synergize™), aldehydes (DC&R®, Synergize™), oxidizing agents (Virkon® S), biguanindes (Nolvasan® Solution), phenol (Tek-Trol®), iodine (Cetridine), chlorine, potassium peroxymonosulfate, phosphate compounds (Stalosan® F), and sulfate compounds effectively inactivate *L. intracellularis*. To allow bacterial inactivation the minimum contact time is 10–30 min in the presence of organic matter and a water hardness of approximately 400 ppm calcium carbonate (43–45). An increase in water hardness (1,000 ppm calcium carbonate) slightly decreases the efficiency of inactivation of a few disinfectants (43).

### Antimicrobials

Utility of antibiotics in feed as a prophylaxis against PE/PHE was appreciated from the early days of identification of the clinical condition (18). With the advent of an *in vitro* culture system for *L. intracellularis*, screening for antimicrobial sensitivity using the rat enterocyte-based culture system (IEC-18; ATCC® CRL-1589™) showed penicillin, erythromycin, difloxacin, virginiamycin, and chlortetracycline had the highest activity to inhibit bacterial multiplication followed by tiamulin and tilmicosin (46). Many antibiotics such as tiamulin, tylosin, tetracycline, lincomycin, and some quinoxalines are documented to be effective at prophylactic doses to control *L. intracellularis* infections (2, 47–54). Years of accumulated evidence indicate that macrolides and pleuromutilins are the most effective prophylaxis for PE/PHE (2). Subtherapeutic levels of antibiotic additives in animal feed have been an integral part of pork production feeding programs for more than 70 years (55). However, in the context of worldwide concerns of antimicrobial resistance, reduction or disuse of prophylactic antimicrobial growth promoters is currently promoted in many parts of the world, including the European Union (Regulation 1831/2003 of the European parliament). Alternatives to control *L. intracellularis* have become a practical requirement.

During clinical disease outbreaks, antibiotics such as tylosin, enrofloxacin, tetracyclines, tiamulin, and tilmicosin are commonly used at higher doses and are effective (2). On the other hand, antimicrobial drugs known to be inherently ineffective against clinical PE/PHE disease outbreaks include penicillin, bacitracin, and aminoglycosides such as neomycin, virginiamycin, and ionophores. In addition, other ineffective antimicrobial therapies include copper or zinc compounds and feed acidifiers (2).

### Vaccines

It has been shown that natural infection with *L. intracellularis* confers robust immunity (56). Specifically, pigs experimentally infected with intestinal mucosal homogenate from naturally infected pigs and treated with antibiotics from day 21 to 31 after infection showed complete resistance to reinfection on day 49, and lack of an acute phase response evidential of natural immunity after primary infection (57, 58). *L. intracellularis* specific cell mediated immunity and IgA in the intestinal lumen were detected in infected pigs (59). Today, commercial live attenuated and inactivated bacterin-based *L. intracellularis* vaccines are available for prophylactic use (Figure 1), each with its own advantages and disadvantages. The live attenuated vaccine, administered orally via the drinking water, drench or liquid feed based on farm specific feeding practices and regional regulations, requires an antibiotic free window 3 days before and after vaccination. Antibiotics do not interfere with the inactivated vaccine but individual pig administration by parenteral route is necessary. Maternal antibodies against *L. intracellularis* typically wane off at 3 weeks, but may persist up to 5 weeks (60). Presence of maternal antibodies after weaning does not interfere with the immune response to live attenuated vaccines, but subsequent protection has not been studied (61). A lag period of 3 to 4 weeks is observed between administration of live attenuated vaccine and development of protective immunity (61). Due to the longer commercial availability of the live attenuated vaccine, more data on this product has been accumulated in the literature. For example, it has been suggested that the immunity induced by the live attenuated *L. intracellularis* vaccine is less efficient than the immunity conferred by pathogenic *L. intracellularis* infection due to the rapid induction of a *L. intracellularis* specific IFNγ–T cell response by the latter (62). Based on correlation of immunoglobulin levels in orally or intramuscularly vaccinated pigs with post-challenge lesions in the ileum and amount of *L. intracellularis* shedding, neutralization is suggested as a major mode of action of live attenuated vaccines (63). Oral, intramuscular, or intraperitoneal route of vaccination with live attenuated vaccine produces similar IgG response (64). However, oral or intraperitoneal administration of the live attenuated vaccine induces a significantly higher IgA response than the intramuscular route of vaccination (64). Besides antibodies, other mechanisms such as cell mediated immunity are also thought to be elicited by the live attenuated vaccine as discussed above (63, 64). Priming of the humoral immune response may be a key mechanism of immunity induced by inactivated vaccines. In summary, observations from the reports above indicate that both humoral and cell mediated immunity are involved in protection against *L. intracellularis* infection, and the quality of immune response induced by vaccine strains of *L. intracellularis* differs from that induced by pathogenic field isolates.

A perceived lack of immunity elicited by the live attenuated vaccine was observed in pigs housed in bedded systems (65). Also, the antibiotic free feed window required for vaccine administration was unwelcome among the veterinarians surveyed (65). Despite a few shortcomings, live attenuated and inactivated *L. intracellularis* vaccines have been proven to be beneficial in many trials, and are known to reduce lesions and shedding associated with *L. intracellularis* infection in challenged pigs (66–71). Furthermore, vaccination also reduces the usage of antibiotics/antimicrobials to control *L. intracellularis* infection (65, 72). However, *L. intracellularis* vaccines do not confer sterile immunity and additional interventions to control PE/PHE may be required (58, 73).

### Other alternatives

Nutritional supplements such as phytogenic ingredients, essential oils, and others are now increasingly used in the control of many animal diseases. Control of necrotic enteritis on the face of disuse of antimicrobial growth promoters in poultry is a prime example of this scenario (74–76). For this condition, caused by the anaerobic bacterium *Clostridium perfringens*, a perceived increase in morbidity and mortality was observed after the removal of antimicrobial growth promoters (75). Many alternate control measures such as prebiotics, probiotics, gut acidifiers, and mannan oligosaccharides are now being used to control necrotic enteritis, which could serve as a model for *L. intracellularis* control (74, 75). Antimicrobial growth promoters such as tylosin are widely used in *L. intracellularis* control (77, 78). They are known to promote a beneficial gut microbiome and a transition to a “mature gut microbiome” (77, 78). Hence, directly manipulating the gut microbiome with feed supplements rather than antimicrobial growth promoters could be a viable approach in *L. intracellularis* control.

### Gut microbiome and *L. intracellularis*

The human gut microbiome is well studied and is known to consist of numerous species of bacteria ranging from 150 up to 400 different species, mostly of Firmicutes, Bacteroidetes, Actinobacteria, and Proteobacteria phyla (79, 80). The bacterial diversity in the human gut is known to be influenced by age, environment, diet content, fiber, cultural factors, host genetics, and other factors (80). Patterns of gut microbiomes in human populations are broadly described as falling under three non-discrete “enterotypes” based on the taxonomic composition (80). Enterotype 1 (or B) is described as dominated by *Bacteroides*, enterotype 2 (or P) is dominated by *Prevotella*, and enterotype 3 (or F) is dominated by Firmicutes, most prominently *Ruminococcus* (80). The *Bacteroides* and *Prevotella* are thought to be inversely correlated in their relative abundance (80). However, other groups suggest dynamic variation of enterotype distribution in individuals in addition to variation due to analysis of proportional data from microbiome studies (81, 82).

Pigs, apart from different dietary regimens, are very similar to humans in their gut physiology and should have similar patterns in gut microbiome development and maintenance. Preliminary studies show that the pig gut microbiome varies from the jejunum to the rectum, with the ileum having a higher relative abundance of Firmicutes (83–87). In the cecum and colon, Firmicutes and Bacteriodetes are comparable in relative abundance and constitute the major phyla (85). Studies also clearly show that the pig gut microbiome changes with age, described as “microbiota succession,” and weaning has an profound impact on the piglets gut microbiome, making them vulnerable to enteric infections (85, 87). Under modern pig production conditions, piglets are weaned at an age of 3–4 weeks, however the natural weaning age of pigs is at 17 weeks and thus weaning is a major stress on the pigs' gastrointestinal tracts (87, 88).

A longitudinal study conducted in 2004 indicates that Danish pigs showed a spurt of *L. intracellularis* shedding 2–3 weeks after weaning, potentially suggesting a role of weaning stress on the onset of *L. intracellularis* infection (89). It is now acknowledged that *L. intracellularis* infection is common in weaned pigs (2). It is also known that *L. intracellularis* infection leads to changes in the pig gut microbiome, indicating a complex association (70, 90, 91). Early studies show that experimental infection of pigs with a cell culture propagated pure *L. intracellularis* inoculum does not cause disease in gnotobiotic pigs but the same inoculum produces disease in conventional pigs (12). In addition, gnotobiotic pigs infected with neomycin treated mucosa from naturally infected pigs showed *L. intracellularis* colonization and disease in the small intestine, suggesting that *L. intracellularis* needs other anaerobic organisms to establish infection (6, 17).

*L. intracellularis* infection is associated with a decrease in mucus secreting goblet cells in the small intestinal epithelium marked by absence of glycoprotein MUC2 expression at the peak of infection (24). Mouse models lacking MUC2 expression are susceptible to intestinal dysbiosis and infections (92). MUC2 knockout mice fed with probiotics show resistance to intestinal dysbiosis and infections (92). On that note, manipulating the gut microbiome with nutritional supplements may be helpful in preventing the establishment of *L. intracellularis* infection and may even assist in mitigating the effect of the pathogen in the face of an infection.

### Nutritional intervention

In recent years, much attention has been given to nutritional supplements, which could potentially aid in controlling *L. intracellularis* intestinal infections. Probiotics are defined as “beneficial” bacteria that may confer a health benefit to the host. On the other hand, prebiotics are food ingredients that induce the growth or activity of probiotics. However, care must be taken in labeling microorganisms as “beneficial,” as available information is often anecdotal or bacterial strain or host species specific. Nutritional changes may potentially be useful in controlling *L. intracellularis* infection if hurdles such as delivery of live organisms to the region of interest and others can be overcome (93).

Prebiotics are prececal non-digestible contents present or added in the feed that act as a substrate for certain intestinal microbes, which in turn produce metabolites (such as short chain fatty acids) and bacteriocins that modulate the gut microbiota, gut morphology, immune system, and other beneficial effects (94–96). In addition, the non-digestible fibers are thought to physically prevent pathogen adhesion to host cells, a mechanism which could potentially prevent the adhesion of *L. intracellularis* to enterocytes. Fructooligosccharides, inulin, and mannanoligosacchrides are some of the well-defined prebiotic feed additives (94). Other resistant starch and complex polysaccharides such as cellulose, hemicellulose, and pectin are known for their prebiotic activity (95, 96). Feed trials in pigs show that insoluble β-glucans present in barley favor an increase in counts of gut bacteria such as *Lactobacillus* spp. and *Bifidobacterium* spp., which are considered beneficial to the gut health (97). Feed texture influences the nature and relative content of intestinal microbiota in pigs. Coarse non-pelleted feed decreases the prevalence of *L. intracellularis* and favors beneficial microbes in experimentally and naturally infected pigs (90, 98). Experimental addition of distiller dried grains with solubles and soybean hulls to pig feed produced a mild mitigation of experimental *L. intracellularis* infection (99–100). Experimental supplementation of short-chain fructooligosaccharide (scFOS) in the sow's feed during the last third of gestation and the entire lactation period resulted in improved general gut immune parameters and *L. intracellularis* specific immune response in their litters (102). These piglets also showed increase in goblet cell number and healthier morphology of intestines compared to the control litters without maternal scFOS feed supplementation. The authors of the above study believe that maternal feeding of scFOS may enhance the beneficial microbiota of the sows which are then transmitted to the piglets, where the microbes increase the production of short chain fatty acids in their gut (102). Another recent study has produced preliminary evidence that feed composition influences *L. intracellularis* infections in farmed pigs (103). Though few specific studies have investigated *L. intracellularis* control by using prebiotics, this approach appears promising and is mediated by the increase in beneficial microbes. In another similar approach, supplementation of natural ingredients with direct antimicrobial properties such as extracts of *Origanum vulgaris* (Oregano) and *Allium sativum* (garlic) in pig feed produced reduction in *L. intracellularis* load in the intestine and a decrease in clinical disease, with improved production parameters in a herd with history of *L. intracellularis* infection (104). Similar effects were observed with a proprietary phytogenic feed additive with essential oils and extracts in field study of a herd naturally exposed to *L. intracellularis* (105). The maximum inclusion level of non-digestible feed ingredients and phytogenic products in the feed is an important consideration to avoid adversely affecting the feed intake and available digestible energy in the feed.

The benefits of fermented food for human consumption, with or without live microbes, in improving gut health and controlling enteric pathogens has been appreciated since ancient times (106, 107). A scientific exposition of health benefits of using *Lactobacillus* to manipulate the human gut microbiome was reported in 1921 (108). *Enterococcus faecium* was shown to suppress diarrhea and mortality induced by pathogenic *Escherichia coli* in pigs (109). In regards to *L. intracellularis*, addition of lactic acid to feed and fermented liquid feed partially mitigated the pathogenesis and shedding of organism in farmed pigs (110). Probiotics are administered as live organisms or spores. They can resist gastric acids and bile, persist in the intestinal tract, produce pathogen inhibitory compounds, elicit an immune response, and alter the gut microbiome composition and activity (111). Certain microbes may elicit indirect benefits by triggering the mucosal innate immune system in the alimentary passage, activating adaptive immune response against antigenic motifs or molecular patterns shared with pathogens, eliciting an anti-inflammatory state in the intestinal epithelial barrier, and enhancing the barrier function of epithelial cells lining the gut (112–115). *Escherichia coli* Nissle, considered a probiotic organism, enhances barrier function of intestinal epithelia and improves protection against rotavirus in pigs; a mechanism which could work in the context of *L. intracellularis* infection (116). Some microbes are also known to directly inhibit pathogenic microbiota by competitive exclusion, secretion of various inhibitory biomolecules, and through enzymes and metabolites; mechanisms potentially useful for control *L. intracellularis* infection (114, 117). Others are known to form biofilms that exclude pathogen colonization (114). Compounds such as polymers of phosphates (PolyP), indole, and competence and sporulation factors produced by *Bacillus spp* are known to elicit beneficial innate immune responses in the gut epithelium and also have systemic effects on the immune system (117). The utility of feed supplements in controlling *L. intracellularis* infection is an avenue left unexplored until now.

## Conclusions

It has been 25 years since *L. intracellularis* was first cultured *in vitro* and 15 years since the genome was sequenced, but understanding of the pathogenesis of this bacterium is still not conclusive. However, beneficial attenuated oral vaccines and inactivated vaccines are available to pig producers. The host response to *L. intracellularis* infection is being gradually unraveled, but the mechanism of *L. intracellularis* pathogenesis and the virulence factors of the bacterium are not yet definitively known. Information obtained from analysis of gene expression patterns of pathogenic and nonpathogenic *L. intracellularis* and infected host cell gene expression profiles need to be further explored. The proliferative lesions observed with clinical *L. intracelluaris* infection are not yet replicated in any *in vitro* infection model. The inability of pure culture of *L. intracellularis* to establish infection in gnotobiotic pigs remains an unexplained observation and would lend clues to the microbial milieu that favors *L. intracellularis* to establish infection. While PPE is observed in weaned and growing pigs and PHE is observed in pigs over 4 months of age, the reason behind this variation in pathogenesis with age is not known. Hamsters, which show susceptibility to the pig *L. intracellularis* and develop pathological lesions similar to that in pigs, could be utilized as a model to further study the disease ecology of *L. intracellularis* under gnotobiotic conditions. Understanding the dynamics between the gut microbiome and *L. intracellularis* infection will help in formulating appropriate diet-based control of the disease, which would be synergistic with current vaccines.

## Author contributions

AK drafted the manuscript and both AK and TO reviewed the literature and revised the final manuscript.

### Conflict of interest statement

The authors declare that the research was conducted in the absence of any commercial or financial relationships that could be construed as a potential conflict of interest.

## References

[1] Ramirez A Differential diagnosis of diseases. In: Zimmerman JJ Karriker LA Ramirez A Schwartz KJ Stevenson GW editors. Diseases of Swine. 10th ed Chichester: Wiley-Blackwell (2012). p. 159–79.

[2] McOrist S Gebhart CJ Proliferative enteropathy. In: Zimmerman JJ Karriker LA Ramirez A Schartz KJ Stevenson GW editors. Diseases of Swine. 10th ed Chichester: Wiley-Blackwell (2012). 811–20.

[3] Biester HE Schwarte LH. Intestinal adenoma in swine. Am J Pathol. (1931) 7:175–85.6. 19969959 PMC2062611

[4] Rowland AC Lawson GHK Maxwell A. Intestinal adenomatosis in the pig: occurance of a bacterium in affected cells. Nature (1973) 243:417. 10.1038/243417a0 4743637

[5] Lawson GHK Rowland AC MacIntyre N. Demonstration of a new intracellular antigen in porcine intestinal adenomatosis and hamster proliferative ileitis. Vet Microbiol. (1985) 10:303–13. 10.1016/0378-1135(85)90001-X 2412336

[6] McOrist S Lawson GH Rowland AC MacIntyre N. Early lesions of proliferative enteritis in pigs and hamsters. Vet Pathol. (1989) 26:260–4. 10.1177/030098588902600311 2669313

[7] Jonas AM Tomita Y Wyand DS. Enzootic intestinal adenocarcinoma in hamsters. J Am Vet Med Assoc. (1965) 147:1102–8. 4956361

[8] Jacoby RO Transmissible ileal hyperplasia of hamsters. I histogenesis and immunocytochemistry Am J Pathol. (1978) 91:433–50. 655259 PMC2018310

[9] Johnson EA Jacoby RO. Transmissible ileal hyperplasia of hamsters. II ultrastructure Am J Pathol. (1978) 91:451–68. 655260 PMC2018313

[10] Stills HFJr. Isolation of an intracellular bacterium from hamsters (*Mesocricetus auratus*) with proliferative ileitis and reproduction of the disease with a pure culture. Infect Immun. (1991) 59:3227–36. 1879939 10.1128/iai.59.9.3227-3236.1991 PMC258157

[11] Lawson GH McOrist S Jasni S Mackie RA. Intracellular bacteria of porcine proliferative enteropathy: cultivation and maintenance *in vitro*. J Clin Microbiol. (1993) 31:1136–42. 8501214 10.1128/jcm.31.5.1136-1142.1993 PMC262892

[12] McOrist S Jasni S Mackie RA MacIntyre N Neef N Lawson GH. Reproduction of porcine proliferative enteropathy with pure cultures of ileal symbiont intracellularis. Infect Immun. (1993) 61:4286–92. 8406817 10.1128/iai.61.10.4286-4292.1993 PMC281156

[13] Jasni S McOrist S Lawson GH. Reproduction of proliferative enteritis in hamsters with a pure culture of porcine ileal symbiont intracellularis. Vet Microbiol. (1994) 41:1–9. 10.1016/0378-1135(94)90130-9 7801512

[14] Gebhart CJ Barns SM McOrist S Lin GF Lawson GH. Ileal symbiont intracellularis, an obligate intracellular bacterium of porcine intestines showing a relationship to *Desulfovibrio* species. Int J Syst Bacteriol. (1993) 43:533–8. 10.1099/00207713-43-3-533 8347512

[15] McOrist S Gebhart CJ Boid R Barns SM. Characterization of *Lawsonia intracellularis* gen. nov, sp nov, the obligately intracellular bacterium of porcine proliferative enteropathy Int J Syst Bacteriol. (1995) 45:820–5. 7547305 10.1099/00207713-45-4-820

[16] Vannucci FA Gebhart CJ. Recent advances in understanding the pathogenesis of *Lawsonia intracellularis* infections. Vet Pathol. (2014) 51:465–77. 10.1177/0300985813520249 24476941

[17] Lawson GH Gebhart CJ. Proliferative enteropathy. J Comp Pathol. (2000) 122:77–100. 10.1053/jcpa.1999.0347 10684678

[18] Love RJ Love DN. Control of proliferative haemorrhagic enteropathy in pigs. Vet Rec. (1977) 100:473. 10.1136/vr.100.22.473 301679

[19] Collins A Love RJ Pozo J Smith HS McOrist S Studies on the *ex vivo* survival of *Lawsonia intracellularis*. Swine Health Prod. (2000) 8:211–5.

[20] Boutrup TS Boesen HT Boye M Agerholm JS Jensen TK. Early pathogenesis in porcine proliferative enteropathy caused by *Lawsonia intracellularis*. J Comp Pathol. (2010) 143:101–9. 10.1016/j.jcpa.2010.01.006 20167332

[21] Guedes RMC Machuca MA Quiroga MA Pereira CER Resende TP Gebhart CJ. *Lawsonia intracellularis* in pigs: progression of lesions and involvement of apoptosis. Vet Pathol. (2017) 54:620–8. 10.1177/0300985817698206 28622490

[22] Jensen TK Christensen BB Boye M. *Lawsonia intracellularis* infection in the large intestines of pigs. APMIS (2006) 114:255–64. 16689824 10.1111/j.1600-0463.2006.apm_53.x

[23] McOrist S Roberts L Jasni S Rowland AC Lawson GH Gebhart CJ. Developed and resolving lesions in porcine proliferative enteropathy: possible pathogenetic mechanisms. J Comp Pathol. (1996) 115:35–45. 8878750 10.1016/s0021-9975(96)80026-0

[24] Huan YW Bengtsson RJ MacIntyre N Guthrie J Finlayson H Smith SH. *Lawsonia intracellularis* exploits beta-catenin/Wnt and Notch signalling pathways during infection of intestinal crypt to alter cell homeostasis and promote cell proliferation. PLoS ONE (2017) 12:e0173782. 10.1371/journal.pone.0173782 28323899 PMC5360247

[25] Oh YS Lee JB McOrist S. Microarray analysis of differential expression of cell cycle and cell differentiation genes in cells infected with *Lawsonia intracellularis*. Vet J. (2010) 184:340–5. 10.1016/j.tvjl.2009.03.018 19362500

[26] Jacobson M Andersson M Lindberg R Fossum C Jensen-Waern M. Microarray and cytokine analyses of field cases of pigs with diarrhoea. Vet Microbiol. (2011) 153:307–14. 10.1016/j.vetmic.2011.06.003 21741782

[27] Vannucci FA Foster DN Gebhart CJ. Laser microdissection coupled with RNA-seq analysis of porcine enterocytes infected with an obligate intracellular pathogen (*Lawsonia intracellularis*). BMC Genomics (2013) 14:421. 10.1186/1471-2164-14-421 23800029 PMC3718617

[28] Smith SH Wilson AD Van Ettinger I MacIntyre N Archibald AL Ait-Ali T. Down-regulation of mechanisms involved in cell transport and maintenance of mucosal integrity in pigs infected with *Lawsonia intracellularis*. Vet Res. (2014) 45:55. 10.1186/1297-9716-45-55 24885874 PMC4031155

[29] Vannucci FA Pusterla N Mapes SM Gebhart C. Evidence of host adaptation in *Lawsonia intracellularis* infections. Vet Res. (2012) 43:53. 10.1186/1297-9716-43-53 22715937 PMC3443049

[30] Sampieri F Vannucci FA Allen AL Pusterla N Antonopoulos AJ Ball KR. Species-specificity of equine and porcine *Lawsonia intracellularis* isolates in laboratory animals. Can J Vet Res. (2013) 77:261–72. 24124268 PMC3788657

[31] Sait M Aitchison K Wheelhouse N Wilson K Lainson FA Longbottom D. (2013). Genome sequence of Lawsonia intracellularis strain N343, isolated from a sow with hemorrhagic proliferative enteropathy. Genome Announc., (2013) 1:e00027–13. 10.1128/genomeA.00027-13 23472224 PMC3587925

[32] Mirajkar NS Kelley MR Gebhart CJ. Draft genome sequence of *Lawsonia intracellularis* Strain E40504, isolated from a horse diagnosed with equine proliferative enteropathy. Genome Announc. (2017) 5:e00330–17. 10.1128/genomeA.00330-17 28495781 PMC5427216

[33] Vannucci FA Kelley MR Gebhart CJ. Comparative genome sequencing identifies a prophage-associated genomic island linked to host adaptation of *Lawsonia intracellularis* infections. Vet Res. (2013) 44–49. 10.1186/1297-9716-44-49 23826661 PMC3716683

[34] Casadesus J Low DA. Programmed heterogeneity: epigenetic mechanisms in bacteria. J Biol Chem. (2013) 288:13929–35. 10.1074/jbc.R113.472274 23592777 PMC3656251

[35] Vannucci FA Foster DN Gebhart CJ. Comparative transcriptional analysis of homologous pathogenic and non-pathogenic *Lawsonia intracellularis* isolates in infected porcine cells. PLoS ONE (2012) 7:e46708. 10.1371/journal.pone.0046708 23056413 PMC3463550

[36] Dale CJ Moses EK Ong CC Morrow CJ Reed MB Hasse D. Identification and sequencing of the groE operon and flanking genes of *Lawsonia intracellularis*: use in phylogeny. Microbiology (1998) 144:2073–84. 10.1099/00221287-144-8-2073 9720028

[37] McCluskey J Hannigan J Harris JD Wren B Smith DG. LsaA, an antigen involved in cell attachment and invasion, is expressed by *Lawsonia intracellularis* during infection *in vitro* and *in vivo*. Infect Immun. (2002) 70:2899–907. 10.1128/IAI.70.6.2899-2907.2002 12010978 PMC128020

[38] Schmitz-Esser S Haferkamp I Knab S Penz T Ast M Kohl C. *Lawsonia intracellularis* contains a gene encoding a functional rickettsia-like ATP/ADP translocase for host exploitation. J Bacteriol. (2008) 190:5746–52. 10.1128/JB.00391-08 18606736 PMC2519521

[39] Alberdi MP Watson E McAllister GE Harris JD Paxton EA Thomson JR. Expression by *Lawsonia intracellularis* of type III secretion system components during infection. Vet Microbiol. (2009) 139:298–303. 10.1016/j.vetmic.2009.06.022 19589649

[40] Watson E Clark EM Alberdi MP Inglis NF Porter M Imrie L. A novel *Lawsonia intracellularis* autotransporter protein is a prominent antigen. Clin Vaccine Immunol. (2011) 18:1282–7. 10.1128/CVI.05073-11 21697340 PMC3147349

[41] Watson E Alberdi MP Inglis NF Lainson A Porter ME Manson E. Proteomic analysis of *Lawsonia intracellularis* reveals expression of outer membrane proteins during infection. Vet Microbiol.(2014) 174:448–55. 10.1016/j.vetmic.2014.10.002 25457368

[42] Kim J Won G Park S Lee JH. Identification of *Lawsonia intracellularis* putative hemolysin protein A and characterization of its immunoreactivity. Vet Microbiol. (2017) 205:57–61. 10.1016/j.vetmic.2017.05.007 28622862

[43] Wattanaphansak S Singer RS Isaacson RE Deen J, Gramm BR, Gebhart CJ. *In vitro* assessment of the effectiveness of powder disinfectant (Stalosan® F) against *Lawsonia intracellularis* using two different assays. Vet Microbiol. (2009) 136:403–7. 10.1016/j.vetmic.2008.12.002. 19144473

[44] Wattanaphansak S Singer RS Gebhart CJ Evaluation of *in vitro* bactericidal activity of commercial disinfectants against *Lawsonia intracellularis*. Swine Health Prod. (2009) 18:11–7.

[45] Collins A Fell S Barchia I Cleaning and disinfection with Virkon-S significantly reduces *Lawsonia intracellularis* survival and transmission to naïve pigs. Swine Health Prod. (2013) 21:144–7.

[46] McOrist S Mackie RA Lawson GH. Antimicrobial susceptibility of ileal symbiont intracellularis isolated from pigs with proliferative enteropathy. J Clin Microbiol. (1995) 33:1314–7. 7615747 10.1128/jcm.33.5.1314-1317.1995 PMC228152

[47] McOrist S Smith SH Shearn MF Carr MM Miller DJ. Treatment and prevention of porcine proliferative enteropathy with oral tiamulin. Vet Rec. (1996) 139:615–8. 9123785

[48] McOrist S Morgan J Veenhuizen MF Lawrence K Kroger HW. Oral administration of tylosin phosphate for treatment and prevention of proliferative enteropathy in pigs. Am J Vet Res. (1997) 58:136–9. 9028475

[49] McOrist S Muller Wager A Kratzer D Sjosten CG. Therapeutic efficacy of water-soluble lincomycin-spectinomycin powder against porcine proliferation enteropathy in a European field study. Vet Rec. (2000) 146:61–5. 10.1136/vr.146.3.61 10674691

[50] Kyriakis SC Bourtzi-Hatzopoulou E Alexopoulos C Kritas SK Polyzopoulou Z Lekkas S. Field evaluation of the effect of in-feed doxycycline for the control of ileitis in weaned piglets. J Vet Med B Infect Dis Vet Public Health (2002) 49:317–21. 10.1046/j.1439-0450.2002.00574.x 12420865

[51] Alexopoulos C Tassis PD Kyriakis CS Tzika ED Papatsiros V Kyriakis SC. First experience on the effect of in-feed lincomycin for the control of proliferative enteropathy in growing pigs. J Vet Med A Physiol Pathol Clin Med. (2006) 53:157–62. 10.1111/j.1439-0442.2006.00803.x 16533333

[52] Guedes RM Franca SA Machado GS Blumer MA da Costa Cruz ECJr. Use of tylvalosin-medicated feed to control porcine proliferative enteropathy. Vet Rec. (2009) 165:342–5. 10.1136/vr.165.12.342 19767637

[53] Wattanaphansak S Singer RS Gebhart CJ. *In vitro* antimicrobial activity against 10 North American and European *Lawsonia intracellularis* isolates. Vet Microbiol. (2009) 134:305–10. 10.1016/j.vetmic.2008.08.007 18823723

[54] Larsen I Nielsen SS Olsen JE Nielsen JP. The efficacy of oxytetracycline treatment at batch, pen and individual level on *Lawsonia intracellularis* infection in nursery pigs in a randomised clinical trial. Prev Vet Med. (2016) 124:25–33. 10.1016/j.prevetmed.2015.12.018 26774445

[55] Li J (2017). Current status and prospects for in-feed antibiotics in the different stages of pork production - a review. Asian-Australas J Anim Sci. 30:1667–73. 10.5713/ajas.17.0418 28823126 PMC5666167

[56] Collins AM Love RJ. Re-challenge of pigs following recovery from proliferative enteropathy. Vet Microbiol. (2007) 120:381–6. 10.1016/j.vetmic.2006.11.004 17188822

[57] Riber U Cordes H Boutrup TS Jensen TK Heegaard PM Jungersen G. Primary infection protects pigs against re-infection with *Lawsonia intracellularis* in experimental challenge studies. Vet Microbiol. (2011) 149:406–14. 10.1016/j.vetmic.2010.11.028 21168983

[58] Riber U Heegaard PM Cordes H Stahl M Jensen TK Jungersen G. Vaccination of pigs with attenuated *Lawsonia intracellularis* induced acute phase protein responses and primed cell-mediated immunity without reduction in bacterial shedding after challenge. Vaccine (2015) 33:156–62. 10.1016/j.vaccine.2014.10.084 25444804

[59] Guedes RM Gebhart CJ. Evidence of cell-mediated immune response and specific local mucosal immunoglobulin (Ig) A production against *Lawsonia intracellularis* in experimentally infected swine. Can J Vet Res. (2010) 74:97–101. 20592838 PMC2851731

[60] Guedes RM Gebhart CJ Armbruster GA Roggow BD. Serologic follow-up of a repopulated swine herd after an outbreak of proliferative hemorrhagic enteropathy. Can J Vet Res. (2002) 66:258–63. 12418781 PMC227013

[61] Walter D Gebhart C Kroll J Holck JT Chittick W Serologic profiling and vaccination timing for *Lawsonia intracellularis*. J Swine Health Prod. (2004) 12:310–31.

[62] Cordes H Riber U Jensen TK Jungersen G. Cell-mediated and humoral immune responses in pigs following primary and challenge-exposure to *Lawsonia intracellularis*. Vet Res. (2012) 43–9. 10.1186/1297-9716-43-9 22316065 PMC3313852

[63] Nogueira MG Collins AM Donahoo M Emery D. Immunological responses to vaccination following experimental *Lawsonia intracellularis* virulent challenge in pigs. Vet Microbiol. (2013) 164:131–8. 10.1016/j.vetmic.2013.02.004 23478250

[64] Nogueira MG Collins AM Dunlop RH Emery D. Effect of the route of administration on the mucosal and systemic immune responses to *Lawsonia intracellularis* vaccine in pigs. Aust Vet J. (2015) 93:124–6. 10.1111/avj.12305 25817978

[65] Holyoake PK Collins A Donahoo M Lising R Emery D. Identifying obstacles to reducing the use of antibiotics to control porcine proliferative enteropathy. Aust Vet J. (2009) 87:33–4. 10.1111/j.1751-0813.2008.00372.x 19178474

[66] Guedes RM Gebhart CJ. Onset and duration of fecal shedding, cell-mediated and humoral immune responses in pigs after challenge with a pathogenic isolate or attenuated vaccine strain of *Lawsonia intracellularis*. Vet Microbiol. (2003) 91:135–45. 10.1016/S0378-1135(02)00301-2 12458163

[67] Kroll JJ Roof MB McOrist S. Evaluation of protective immunity in pigs following oral administration of an avirulent live vaccine of *Lawsonia intracellularis*. Am J Vet Res. (2004) 65:559–65. 10.2460/ajvr.2004.65.559 15141873

[68] Almond PK Bilkei G. Effects of oral vaccination against *Lawsonia intracellularis* on growing-finishing pig's performance in a pig production unit with endemic porcine proliferative enteropathy (PPE). Dtsch Tierarztl Wochenschr (2006) 113:232–5. 16856610

[69] McOrist S Smits RJ. Field evaluation of an oral attenuated *Lawsonia intracellularis* vaccine for porcine proliferative enteropathy (ileitis). Vet Rec. (2007) 161:26–8. 10.1136/vr.161.1.26 17617542

[70] Leite FLL Singer RS Ward T Gebhart CJ Isaacson RE. Vaccination against *Lawsonia intracellularis* decreases shedding of *Salmonella enterica* serovar Typhimurium in co-infected pigs and alters the gut microbiome. Sci Rep. (2018) 8–2857. 10.1038/s41598-018-21255-7 29434295 PMC5809363

[71] Roerink F Morgan CL Knetter SM Passat MH Archibald AL Ait-Ali T. A novel inactivated vaccine against *Lawsonia intracellularis* induces rapid induction of humoral immunity, reduction of bacterial shedding and provides robust gut barrier function. Vaccine (2018) 36:1500–8. 10.1016/j.vaccine.2017.12.049 29336925 PMC5846845

[72] Bak H Rathkjen PH. Reduced use of antimicrobials after vaccination of pigs against porcine proliferative enteropathy in a Danish SPF herd. Acta Vet Scand. (2009) 51–1. 10.1186/1751-0147-51-1 19128459 PMC2633004

[73] Kruse AB de Knegt LV Nielsen LR Alban L. No clear effect of initiating vaccination against common endemic infections on the amounts of prescribed antimicrobials for Danish weaner and finishing pigs during 2007-2013. Front Vet Sci. (2016) 3–120. 10.3389/fvets.2016.00120 28138438 PMC5237653

[74] Caly DL D'Inca R Auclair E Drider D. Alternatives to antibiotics to prevent necrotic enteritis in broiler chickens: a microbiologist's perspective. Front Microbiol. (2015) 6–1336. 10.3389/fmicb.2015.01336 26648920 PMC4664614

[75] M'Sadeq SA Wu S Swick RA Choct M. Towards the control of necrotic enteritis in broiler chickens with in-feed antibiotics phasing-out worldwide. Anim Nutr. (2015) 1:1–11. 10.1016/j.aninu.2015.02.004 29766984 PMC5884463

[76] Prescott JF Parreira VR Mehdizadeh Gohari I Lepp D Gong J. The pathogenesis of necrotic enteritis in chickens: what we know and what we need to know: a review. Avian Pathol. (2016) 45:288–94. 10.1080/03079457.2016.1139688 26813023

[77] Kim HB Borewicz K White BA Singer RS Sreevatsan S Tu ZJ. Microbial shifts in the swine distal gut in response to the treatment with antimicrobial growth promoter, tylosin. Proc Natl Acad Sci USA. (2012) 109:15485–90. 10.1073/pnas.1205147109 22955886 PMC3458334

[78] Kim J Guevarra RB Nguyen SG Lee JH Jeong DK Unno T. Effects of the antibiotics growth promoter Tylosin on swine gut microbiota. J Microbiol Biotechnol. (2016) 26:876–82. 10.4014/jmb.1512.12004 26869601

[79] Davenport ER Sanders JG Song SJ Amato KR Clark AG Knight R. The human microbiome in evolution. BMC Biol. (2017) 15–127. 10.1186/s12915-017-0454-7 29282061 PMC5744394

[80] Costea PI Hildebrand F Arumugam M Backhed F Blaser MJ Bushman FD Enterotypes in the landscape of gut microbial community composition. Nat Microbiol. (2018) 3:8–16. 10.1038/s41564-017-0072-8 29255284 PMC5832044

[81] Knights D Ward TL McKinlay CE Miller H Gonzalez A McDonald D. Rethinking “enterotypes”. Cell Host Microbe. (2014) 16:433–7. 10.1016/j.chom.2014.09.013 25299329 PMC5558460

[82] Sze MA Schloss PD. Looking for a signal in the noise: revisiting obesity and the microbiome. MBio (2016) 7:e01018–16. 10.1128/mBio.01018-16. 27555308 PMC4999546

[83] Isaacson R Kim HB. The intestinal microbiome of the pig. Anim Health Res Rev. (2012) 13:100–9. 10.1017/S1466252312000084 22853934

[84] Frese SA Parker K Calvert CC Mills DA. Diet shapes the gut microbiome of pigs during nursing and weaning. Microbiome (2015) 3–28. 10.1186/s40168-015-0091-8 26167280 PMC4499176

[85] Kim HB Isaacson RE. The pig gut microbial diversity: understanding the pig gut microbial ecology through the next generation high throughput sequencing. Vet Microbiol. (2015) 177:242–51. 10.1016/j.vetmic.2015.03.014 25843944

[86] Mach N Berri M Estelle J Levenez F Lemonnier G Denis C. Early-life establishment of the swine gut microbiome and impact on host phenotypes. Environ Microbiol Rep. (2015) 7:554–69. 10.1111/1758-2229.12285 25727666

[87] Gresse R Chaucheyras-Durand F Fleury MA Van de Wiele T Forano E Blanquet-Diot S. Gut microbiota dysbiosis in postweaning piglets: understanding the keys to health. Trends Microbiol. (2017) 25:851–73. 10.1016/j.tim.2017.05.004 28602521

[88] Jensen P Observations on the maternal behaviour of free-ranging domestic pigs. Appl Anim Behav Sci. (1986) 16:131–42. 10.1016/0168-1591(86)90105-X

[89] Stege H Jensen TK Moller K Vestergaard K Baekbo P Jorsal SE. Infection dynamics of *Lawsonia intracellularis* in pig herds. Vet Microbiol. (2004) 104:197–206. 10.1016/j.vetmic.2004.09.015 15564028

[90] Molbak L Johnsen K Boye M Jensen TK Johansen M Moller K. The microbiota of pigs influenced by diet texture and severity of *Lawsonia intracellularis* infection. Vet Microbiol. (2008) 128:96–107. 10.1016/j.vetmic.2007.09.012 17996403

[91] Borewicz KA Kim HB Singer RS Gebhart CJ Sreevatsan S Johnson T. Changes in the porcine intestinal microbiome in response to infection with *Salmonella enterica* and *Lawsonia intracellularis*. PLoS ONE (2015) 10:e0139106. 10.1371/journal.pone.0139106 26461107 PMC4604083

[92] Kumar M Kissoon-Singh V Coria AL Moreau F Chadee K Probiotic mixture VSL#3 reduces colonic inflammation and improves intestinal barrier function in Muc2 mucin-deficient mice. Am J Physiol Gastrointest Liver Physiol. (2017) 312:G34–45. 10.1152/ajpgi.00298.2016 27856417

[93] Barba-Vidal E Martín-Orúe SM Castillejos L Review: are we using probiotics correctly in post-weaning piglets? Animal (2018) 3:1–10. 10.1017/S1751731118000873 29720287

[94] Pourabedin M Zhao X. Prebiotics and gut microbiota in chickens. FEMS Microbiol Lett. (2015) 362:fnv122. 10.1093/femsle/fnv122 26208530

[95] Holscher HD. Dietary fiber and prebiotics and the gastrointestinal microbiota. Gut Microbes (2017) 8:172–84. 10.1080/19490976.2017.1290756 28165863 PMC5390821

[96] Umu OCO Rudi K Diep DB. Modulation of the gut microbiota by prebiotic fibres and bacteriocins. Microb Ecol Health Dis. (2017) 28–1348886. 10.1080/16512235.2017.1348886 28959178 PMC5614387

[97] Murphy P Bello FD O'Doherty JV Arendt EK Sweeney T Coffey A. Effects of cereal beta-glucans and enzyme inclusion on the porcine gastrointestinal tract microbiota. Anaerobe (2012) 18:557–65. 10.1016/j.anaerobe.2012.09.005 23022204

[98] Stege H Jensen TK Moller K Baekbo P Jorsal SE. Risk factors for intestinal pathogens in Danish finishing pig herds. Prev Vet Med. (2001) 50:153–64. 10.1016/S0167-5877(01)00194-5 11448502

[99] Whitney MH Shurson GC Guedes RC. Effect of including distillers dried grains with solubles on the ability of growing pigs to resist a *Lawsonia intracellularis* challenge. J Anim Sci. (2006) 84:1860–8. 10.2527/jas.2004-574 16775070

[100] Whitney MH Shurson GC Guedes RC. Effect of dietary inclusion of distillers dried grains with solubles, soybean hulls, or a polyclonal antibody product on the ability of growing pigs to resist a *Lawsonia intracellularis* challenge. J Anim Sci. (2006) 84:1880–9. 10.2527/jas.2004-578 16775072

[101] Whitney MH Shurson GC Guedes RC. Effect of including distillers dried grains with solubles in the diet, with or without antimicrobial regimen, on the ability of growing pigs to resist a *Lawsonia intracellularis* challenge. J Anim Sci. (2006) 84:1870–9. 10.2527/jas.2004-575 16775071

[102] Le Bourgot C Le Normand L Formal M Respondek F Blat S Apper E Maternal short-chain fructo-oligosaccharide supplementation increases intestinal cytokine secretion, goblet cell number, butyrate concentration and *Lawsonia intracellularis* humoral vaccine response in weaned pigs. Br J Nutr. (2017) 117:83–92. 10.1017/S0007114516004268 28115029

[103] Visscher C Kruse A Sander S Keller C Mischok J Tabeling R. Experimental studies on effects of diet on *Lawsonia intracellularis* infections in fattening boars in a natural infection model. Acta Vet Scand. (2018) 60–22. 10.1186/s13028-018-0378-4 29650043 PMC5898007

[104] Papatsiros VG Tzika ED Papaioannou DS Kyriakis SC Tassis PD Kyriakis CS. Effect of *Origanum vulgaris* and *Allium sativum* extracts for the control of proliferative enteropathy in weaning pigs. Pol J Vet Sci. (2009) 12:407–14. 19886265

[105] Draskovic V Bosnjak-Neumuller J Vasiljevic M Petrujkic B Aleksic N Kukolj V. Influence of phytogenic feed additive on *Lawsonia intracellularis* infection in pigs. Prev Vet Med. (2018) 151:46–51. 10.1016/j.prevetmed.2018.01.002 29496105

[106] Fuller R. Probiotics in man and animals. J Appl Bacteriol. (1989) 66:365–78. 10.1111/j.1365-2672.1989.tb05105.x 2666378

[107] Fuller R. Probiotics in human medicine. Gut (1991) 32:439–42. 10.1136/gut.32.4.439 1902810 PMC1379087

[108] Rettger LF Cheplin H Treatise on the Transformation of the Intestinal Flora With Special Reference to the Implantation of Bacillus acidophilus. New Haven, Connecticut: Yale University Press (1921).

[109] Underdahl NR. The effect of feeding *Streptococcus faecium* upon *Escherichia coli* induced diarrhea in gnotobiotic pigs. Prog Food Nutr Sci. (1983) 7:5–12. 6361858

[110] Boesen HT Jensen TK Schmidt AS Jensen BB Jensen SM Moller K. The influence of diet on *Lawsonia intracellularis* colonization in pigs upon experimental challenge. Vet Microbiol. (2004) 103:35–45. 10.1016/j.vetmic.2004.06.008 15381264

[111] Patterson JA Burkholder KM. Application of prebiotics and probiotics in poultry production. Poult Sci. (2003). 82:627–31. 10.1093/ps/82.4.627 12710484

[112] Lalles JP. Microbiota-host interplay at the gut epithelial level, health and nutrition. J Anim Sci Biotechnol. (2016) 7–66. 10.1186/s40104-016-0123-7 27833747 PMC5101664

[113] Reid G. Probiotics: definition, scope and mechanisms of action. Best Pract Res Clin Gastroenterol. (2016) 30:17–25. 10.1016/j.bpg.2015.12.001 27048893

[114] Chiu L Bazin T Truchetet ME Schaeverbeke T Delhaes L Pradeu T. Protective microbiota: from localized to long-reaching co-immunity. Front Immunol. (2017) 8–1678. 10.3389/fimmu.2017.01678 29270167 PMC5725472

[115] Lebeer S Bron PA Marco ML Van Pijkeren JP O'Connell Motherway M Hill C. Identification of probiotic effector molecules: present state and future perspectives. Curr Opin Biotechnol. (2018) 49:217–23. 10.1016/j.copbio.2017.10.007 29153882

[116] Kandasamy S Vlasova AN Fischer DD Chattha KS Shao L Kumar A. Unraveling the Differences between gram-positive and gram-negative probiotics in modulating protective immunity to enteric infections. Front Immunol. (2017) 8–334. 10.3389/fimmu.2017.00334 28396664 PMC5366325

[117] Ilinskaya ON Ulyanova VV Yarullina DR Gataullin IG. Secretome of intestinal bacilli: a natural guard against pathologies. Front Microbiol. (2017) 8–1666. 10.3389/fmicb.2017.01666 28919884 PMC5586196

